# Overcoming Mn-induced chlorosis in sugarcane seedlings by iron

**DOI:** 10.3389/fpls.2025.1739211

**Published:** 2026-01-22

**Authors:** Dongling Li, Guizhi Ling, Shu Yang

**Affiliations:** 1 State Key Laboratory for Conservation and Utilization of Subtropical Agro-bioresources, Guangxi University, Nanning, China; 2 School for Environment and Sustainability, University of Michigan, Ann Arbor, MI, United States

**Keywords:** sugarcane, Mn-induced chlorosis, greening, strategies, iron nutrition

## Abstract

**Background:**

Manganese (Mn) toxicity induces severe seedling chlorosis and growth inhibition in sugarcane cultivated on acidic soils, yet the mechanisms driving seasonal recovery and scalable mitigation strategies remain poorly defined. This study aimed to elucidate the role of iron (Fe) deposition from rainwater in natural greening and validate foliar Fe supplementation as an efficient countermeasure.

**Methods:**

We integrated field monitoring across 78 sites, phenological tracking of seasonal recovery, molecular analyses of chlorophyll biosynthesis pathways, and validation experiments (hydroponic and field trials) to investigate Fe-mediated Mn toxicity alleviation. Key metrics included leaf chlorophyll/Fe correlations, gene expression patterns, and agronomic responses to Fe treatments.

**Results:**

Field data revealed a strong positive correlation between leaf chlorophyll content and foliar Fe levels (r=0.82, p<0.01). Chlorotic seedlings achieved full visual recovery by late summer, with chlorophyll and Fe concentrations increasing 11.1- and 4.4-fold relative to spring baselines. Mechanistically, Fe reversed Mn-induced functional Fe deficiency by enhancing 5-aminolevulinic acid synthesis (2.3-fold increase) and Mg-protoporphyrin IX monomethyl ester conversion (1.8-fold increase), while downregulating *FLUORESCENT* expression (60% reduction) and upregulating MgPME cyclase activity (3.1-fold increase). Foliar FeSO₄ applications (0.5–1.5 g Fe L⁻¹) effectively reversed chlorosis, boosting chlorophyll content by 1.9–2.7-fold, seedling survival by 100%, and cane yield by 1.7-fold under Mn-toxic conditions, with minimal input requirements (7.5–22.5 g Fe ha⁻¹).

**Conclusion:**

Our findings demonstrate that rainwater-borne Fe is a key driver of seasonal recovery from Mn-induced chlorosis in sugarcane. Foliar Fe supplementation emerges as a cost-effective, scalable strategy for mitigating Mn toxicity, offering significant advantages over resource-intensive soil amendments for sustainable sugarcane production on acidic soils.

## Introduction

Soil acidification is a major global constraint to agricultural productivity, with acidic soils (pH ≤ 5.5) occupying over 50% of the world’s arable land, particularly in tropical and subtropical regions (Wang et al., 2023). In China, approximately 32% of the total land area is acidic, mainly in the south (Zhao et al., 2023). Since the 1980s, soil pH in major crop-production regions has declined by 0.13–0.80 units, with more severe acidification observed in the leached red and yellow soils of southern China (Guo et al., 2010; Wang et al., 2023). When soil pH drops below 5.5, divalent manganese ions (Mn^2+^) are released into the soil solution, becoming a major constraint to plant growth and crop production (Horst, 2012; Barker and Pilbeam, 2015). Excessive Mn interferes with the uptake, translocation, and utilization of other essential elements, inhibits enzyme activity, induces oxidative stress, and ultimately reduces photosynthesis and plant growth (Barker and Pilbeam, 2015; Zhu et al., 2025). Mn toxicity causes visible leaf chlorosis in maize (*Zea mays L*.) (Stoyanova et al., 2009), bush bean (*Phaseolus vulgaris* L.) (Horiguchi, 1988), and barley (*Hordeum vulgare* L.) (Huang et al., 2018), and is associated with chlorophyll (Chl) breakdown due to photobleaching and/or photooxidative damage to chloroplasts in common bean (Huang et al., 2018; Gonzalez et al., 1998), sugar maple (*Acer saccharum* Marsh.), and red maple (*Acer rubrum* L.) (St Clair and Lynch, 2004).

Iron (Fe), the most abundant transition metal in soils, commonly coexists with Mn in soil minerals and becomes more soluble under acidic conditions. The well-documented antagonistic interaction between Fe and Mn arises from their similar ionic radii, competition for shared transport systems, analogous chemistry, and partially overlapping physiological functions (Barker and Pilbeam, 2015; Shao et al., 2017; Yang et al., 2022). Previous studies have shown that Fe supply can mitigate Mn toxicity by reducing tissue Mn accumulation in Arabidopsis (*Arabidopsis thaliana* L.) (Yang et al., 2022) and soybean (*Glycine max* L.) (Van Der Vorm and Van Diest, 1979), restoring Mn-impaired Fe metabolism in soybean (Yi et al., 2022), alleviating Mn-induced chlorosis in barley (Huang et al., 2016), soybean, and sunflower (*Helianthus annuus* L.) (Mehrotra and Gupta, 1990; Shen et al., 2017), and enhancing Mn tolerance in Arabidopsis (Yang et al., 2022). However, Fe–Mn interactions vary among species. In barley and tomato (*Solanum lycopersicum* L.), Fe supply does not significantly alter tissue Mn concentrations (Yi et al., 2022; Foy et al., 1998), and in rice (*Oryza sativa* L.) Fe application has no effect on Mn uptake (Van Der Vorm and Van Diest, 1979). These contrasting responses may reflect differences in experimental conditions (e.g., pH, Fe/Mn ratios) or species-specific adaptations in Mn homeostasis and detoxification.

Sugarcane (*Saccharum* spp. hybrids), an important tropical crop for sugar, fiber, and biofuel production (Yi et al., 2022), is often grown on acidic soils where Mn toxicity frequently causes severe leaf chlorosis and markedly reduces yield (Yang et al., 2022; Huang et al., 2016). Our previous mechanistic studies indicate that Mn toxicity in sugarcane primarily impairs chlorophyll biosynthesis through dual inhibition of ALA synthesis and MgPME conversion, rather than by accelerating chlorophyll degradation (Yang et al., 2022). Field observations also show that chlorotic seedlings can recover greenness during late-summer rainfall, suggesting an environment-dependent recovery mechanism. This study therefore aimed to (1) elucidate the physiological mechanisms underlying rain-associated greening in Mn-stressed sugarcane, (2) determine the role of Fe in this process, and (3) assess whether these mechanisms can inform the development of highly effective agronomic strategies to mitigate Mn-induced chlorosis under field conditions. We hypothesized that rainwater-derived Fe facilitates seasonal recovery by restoring chlorophyll biosynthesis, and that foliar Fe supplementation can replicate this effect under field conditions.

## Materials and methods

### Field surveys

To assess whether Mn-induced chlorosis in sugarcane is associated with foliar Fe nutrition, field surveys were conducted during March–April 2014 in six major sugarcane-producing regions of Guangxi, China (Chongzuo, Laibin, Nanning, Liuzhou, Yizhou, and Guigang), located within 22˚33ˊ-22˚43ˊN latitude and 107˚31ˊ-109°24ˊE longitude, which together account for over 50% of national sugarcane cultivation. Soils in these areas are strongly acidic (pH 3.6–4.8) and consistently induce seedling-stage chlorosis (Martínez-Cuenca et al., 2013). In each of 78 cultivation plots, the first fully expanded leaves of sugarcane cv. XTT22 were collected from 30-day-old seedlings. From each plot, 20 plants were randomly selected, and their leaves were pooled to form one composite sample, with three biological replicates per plot (total n = 234). Samples were placed in ice-cooled insulated containers and transported to the laboratory for Fe and chlorophyll (Chl) analyses.

Seasonal changes in leaf Fe concentration during chlorosis recovery were monitored in three geographically separated plots in Quli Town, Fusui County (22˚59N, 107˚584ˊE), Guangxi, in 2016. Severely chlorotic seedlings (SPAD < 4) were sampled at six time points: day 0 (April 1), 5, 15, 25, 35, and 60 after identification. The first fully expanded leaves were analyzed for active Fe and Mn concentrations, and non-destructive Chl measurements were taken from 20 randomly selected plants per plot using a SPAD-502 Plus meter (Konica Minolta, Japan).

### Culture experiments

A hydroponic experiment was conducted to evaluate the role of rainwater in alleviating Mn-induced chlorosis. Ratoon seedlings (cv. Guitang 32) were regenerated from parent plants pre-treated with 0.5 mM MnCl_2_ in one-fifth-strength Hoagland solution (pH 5.5) for 30 days under controlled growth chamber conditions (28°C, 16/8 h light/dark). Chlorotic seedlings (15 days old) were transplanted into 5.5-L plastic pots (4 seedlings per pot) containing aerated Fe-free one-fifth-strength Hoagland solution prepared with either filtered rainwater or deionized water (control), with or without foliar spraying (three times daily) of the corresponding water. Rainwater was collected in May 2018 using acid-washed polyethylene containers. The collected rainwater exhibited the following basic properties: pH 5.91, Fe (0.26 mg L^−1^), Ca (0.74 mg L^−1^), Mg (0.03 mg L^−1^), K (0.11 mg L^−1^), NO_3_^-^ (0.77 mg L^−1^), SO_4_^2−^ (1.30 mg L^−1^), and Cl^−^ (0.17 mg L^−1^). After 15 days, the first fully expanded leaves were photographed, analyzed for Chl concentration (SPAD-502 meter), and harvested for Fe and Mn determination.

To examine the effect of Fe supplementation, seedlings (cv. Guitang 32) prepared as described by Yang (Yang et al., 2022) were cultivated in 5.5-L plastic pots (4 seedlings per pot) and exposed to one of three treatments based on an Fe-free one-fifth-strength Hoagland solution (pH 5.5): (1) control (Fe-free solution), (2) 0.5 mM Mn (MnCl_2_), and (3) 0.5 mM Mn + 10 μM Fe (supplied with EDTA-Fe). Fifteen days after treatment, the first fully expanded leaves were analyzed for Chl, 5-aminolevulinic acid (ALA), Mg-protoporphyrin IX monomethyl ester (MgPME), Mg-protoporphyrin IX (MgProto), protochlorophyllide (Pchlide), and expression levels of *FLUORESCENT (FLU*) and MgPME cyclase (*MgPMEC*).

### Field experiment

Field trials were conducted in 2024 at a sugarcane plantation (22°41′N, 107°48′E) located in Quli Town, Chongzuo City, Guangxi, China, on acidic lateritic soil with naturally high Mn bioavailability. The soil was developed from Quaternary red clay parent material. It was characterized by the following basic properties: pH 5.1, organic matter content of 18.78 g kg^−1^, total nitrogen content of 1.14 g kg^−1^, available phosphorus content of 41.0 mg kg^−1^, available potassium content of 193.0 mg kg^−1^, and available manganese content of 321.0 mg kg^−1^. Sugarcane (cv. Guitang 32) was planted in 2023 at a density of 105,000 buds per hectare. Similar experiments were also conducted during the 2022 and 2023 growing seasons at three additional field sites, and the 2024 trial is presented here as a representative example. These trials employed a randomized complete block design with plots each covering an area of 100 m² and three biological replicates per treatment. On May 9, seedlings exhibiting severe Mn toxicity (interveinal chlorosis, high leaf Mn concentration, and reduced Chl concentration) were foliar-sprayed with FeSO_4_ solution at four Fe concentrations: 0 (control), 0.5, 1.0, or 1.5 g L^−1^. The spray was applied at a rate of 750 kg ha^-1^. Leaf appearance was documented at 0, 3, 13, and 33 days after treatment (DAT). The first fully expanded leaves were sampled for Fe and Chl analyses, and seedling survival was recorded at 13 DAT. At maturity, cane yield was measured through plot-wide stalk harvesting, weighing, and yield calculation.

### Determination of Fe and Mn concentrations

Fe and Mn concentrations were measured by flame atomic absorption spectrometry (PinAAcle 900T, PerkinElmer) (Yang et al., 2022). Fresh leaves (~1 mm fragments) were prepared using stainless steel scissors; dried leaves were oven-dried at 70°C to constant weight, ground, digested with concentrated HNO_3_ at 140°C, and analyzed for total Fe and Mn (Yang et al., 2022). Rainwater Fe concentration was measured after filtration. Active Fe in fresh leaves was extracted by immersion in 1 M HCl for 24 h with occasional shaking, filtered, and quantified spectrophotometrically at 510 nm (UV2600, Shimadzu, Japan) using ortho-phenanthroline as the chelating agent (Mehrotra and Gupta, 1990).

### Determination of chlorophyll concentration

Chl was extracted from fresh leaves with 80% (v/v) acetone in the dark until complete bleaching. Absorbance was measured at 645 and 663 nm (UV2600, Shimadzu, Japan), and total Chl concentration was calculated following Yang et al. (2022).

### Determination of Proto, MgProto, and Pchlide

Proto, MgProto, and Pchlide were determined as described by Yang et al. (2022). Fresh leaves were ground in liquid nitrogen, extracted in 80% alkaline acetone (v/v, alkalized with 0.1 M NH_4_OH), and incubated in the dark until bleaching. After centrifugation (15,000 ×g, 10 min, 4°C), absorbance of the supernatant was measured at 575, 590, and 628 nm, and pigment concentrations were calculated as previously described (Shen et al., 2017).

### Gene expression analysis

Upon termination of the experiments, the first expanded leaves were collected, immediately frozen in liquid nitrogen, and stored at -80°C until analysis. Total RNA was extracted from the leaves, followed by cDNA synthesis and quantitative real-time PCR (qRT-PCR); gene expression levels were then determined using gene-specific primers, all according to the methods described by Yang et al. (2022). The *β*-actin gene was used as a reference gene for gene expression data normalization.

### Statistical analysis

Results were analyzed by using analysis of variance. Where the F-test indicated significant differences (P ≤ 0.05), treatment means were compared by Duncan’s multiple range test. All analyses and graphical presentations were performed using Origin Pro 2019.

## Results

### Relationship between leaf Fe and chlorophyll concentration

**Figure 1:**
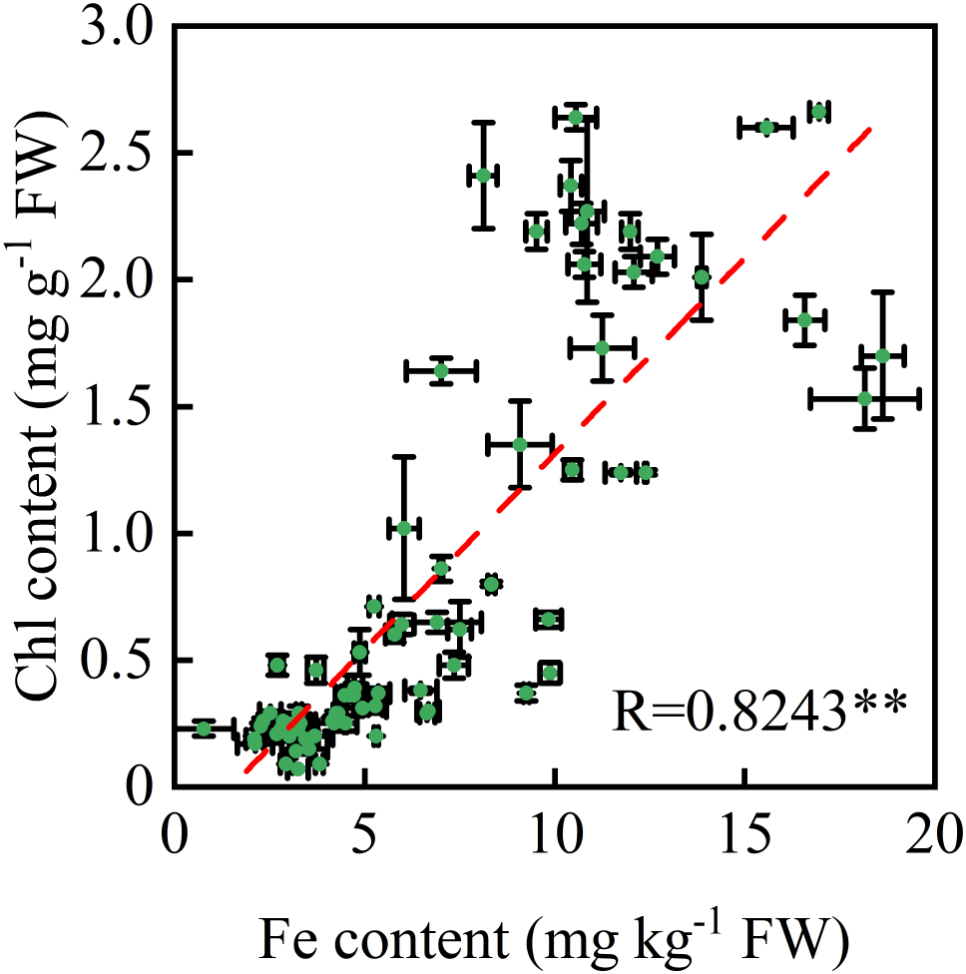
Relationship between leaf iron (Fe) concentration and chlorophyll content in sugarcane seedlings grown on acidic soils (pH 3.2–5.5). Samples were collected from 78 independent field plots during March–April. Each point represents a composite sample of 20 pooled leaves, with three biological replicates per plot. Values are means ± SE (n = 3). ** indicate a significant Pearson correlation (p<0.01).

Mn-induced chlorosis in sugarcane seedlings grown on acidic soils was confirmed in Guangxi, China (**Supplementary Figure 1A**). During the dry spring, affected seedlings exhibited interveinal chlorosis; however, surviving plants progressively regained greenness, with complete symptom remission by the rainy summer season (**Supplementary Figures 1B, C**). Across five major production cities, a strong positive correlation was observed between leaf Fe and chlorophyll (Chl) concentrations (r = 0.82, p < 0.01; **Figure 1**). Elevated leaf Fe concentrations were consistently associated with higher Chl levels under Mn stress, suggesting a role for Fe in mitigating Mn-induced chlorosis.

### Temporal dynamics of leaf Fe during chlorosis recovery

**Figure 2:**
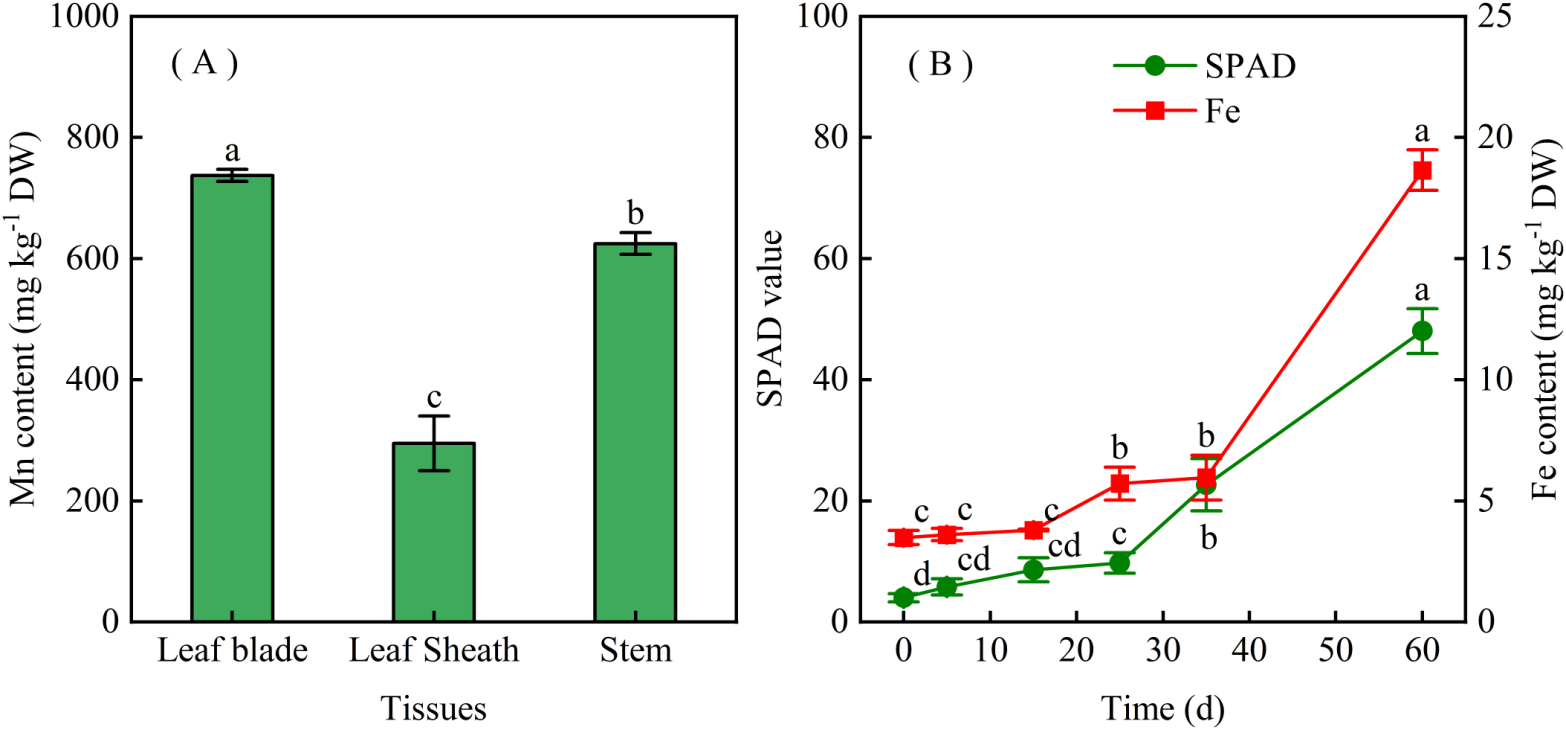
Seasonal changes in **(A)** leaf Fe concentration and **(B)** SPAD values in sugarcane seedlings grown on strongly acidic soil. Leaves were sampled on April 1 (day 0) and subsequently at the indicated time points. Values are means ± SE (n = 3). Significant differences in **(A)** leaf Fe concentration among tissue types were determined by one-way ANOVA, while differences in **(B)** SPAD values across sampling dates were analyzed by repeated-measures one-way ANOVA. For analyses showing significant overall effects, *post hoc* comparisons were performed using Duncan’s test. Prior to ANOVA, SPAD data were subjected to arcsine transformation. Different lowercase letters indicate statistically significant differences (p < 0.05).

In 2016 field monitoring plots, natural recovery of chlorotic seedlings began in mid-May, with full greenness restored in newly emerged leaves by early June. Mn concentrations in all tissues exceeded established toxicity thresholds (Barker and Pilbeam, 2015), with mean values of 767.5 ± 9.9 mg kg^−1^ DW (leaf blade), 295.1 ± 4.5 mg kg^−1^ DW (leaf sheath), and 624.7 ± 17.9 mg kg^−1^ DW (stem) (**Figure 2A**). Leaf SPAD values increased by 71% at 35 days after observation (DAO) and by 4.4-fold at 60 DAO relative to chlorotic baselines (**Figure 2B**), coinciding with complete symptom disappearance (SPAD > 48.1 ± 3.7). Leaf Fe concentration increased 0.7-fold at 35 DAO and 4.4-fold at 60 DAO, supporting the association between Fe accumulation and chlorosis recovery.

### Rainwater alleviates Mn-induced chlorosis

**Figure 3:**
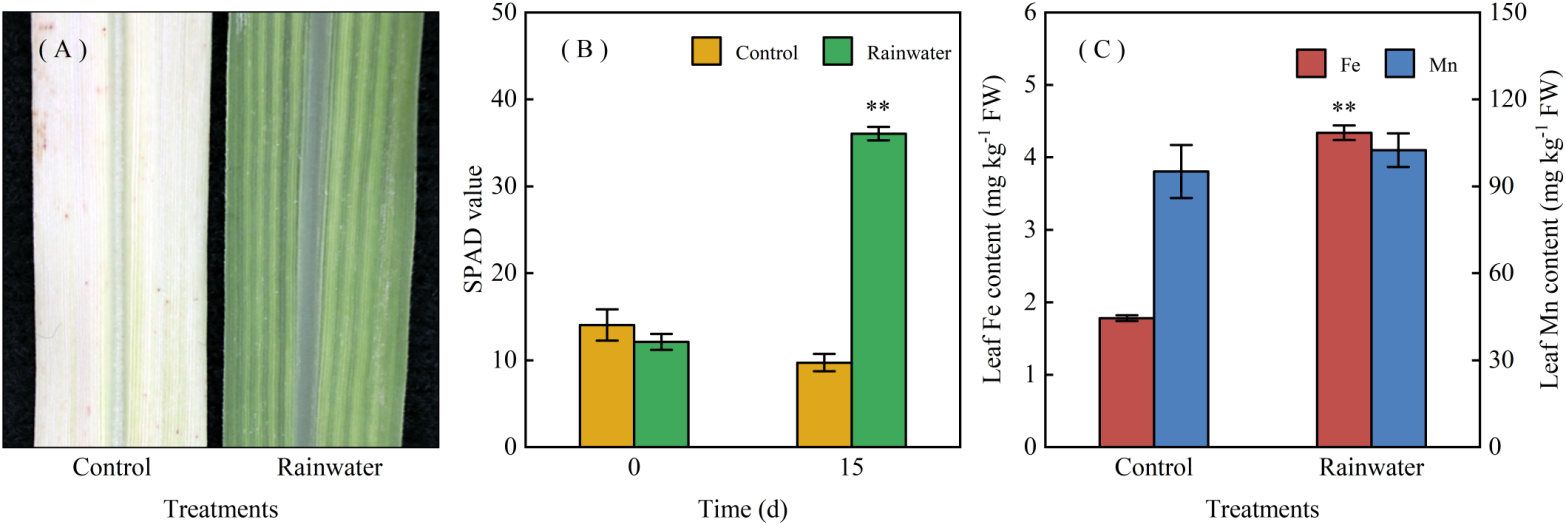
Effects of rainwater application on Mn-induced chlorotic sugarcane seedlings. **(A)** representative leaves at 15 days after treatment (DAT); **(B)** SPAD values at 0 and 15 DAT; **(C)** leaf Fe and Mn concentrations at 15 DAT. Values are means ± SE (n = 3). **above the bars indicate a significant difference at *p*<0.01 (Student’s t-test).

Long-term field observations (since 2010) showed that chlorotic seedlings recovered following the onset of the rainy season. Under controlled conditions, rainwater application—via both foliar spray and root-zone exposure—restored greenness within 15 days (**Figure 3A**), increasing SPAD values by 2.7-fold compared with baseline (p < 0.01; **Figure 3B**). In contrast, deionized water controls developed progressive albinism with no SPAD change (**Figures 3A, B**). These results demonstrate that rainwater is sufficient to trigger greening in Mn-stressed sugarcane seedlings.

### Rainwater supplies bioavailable Fe to chlorotic leaves

Rainwater application increased leaf Fe concentration by 1.4-fold compared with the deionized water control (p < 0.01; **Figure 3C**), without affecting Mn concentration. Rainwater contained an average of 0.26 mg L^−1^ Fe, indicating a direct Fe supply to chlorotic leaves. The lack of recovery in Fe-free controls confirms that rainwater-derived Fe is essential for alleviating Mn-induced chlorosis.

### Fe restores chlorophyll biosynthesis in Mn-stressed seedlings

**Figure 4:**
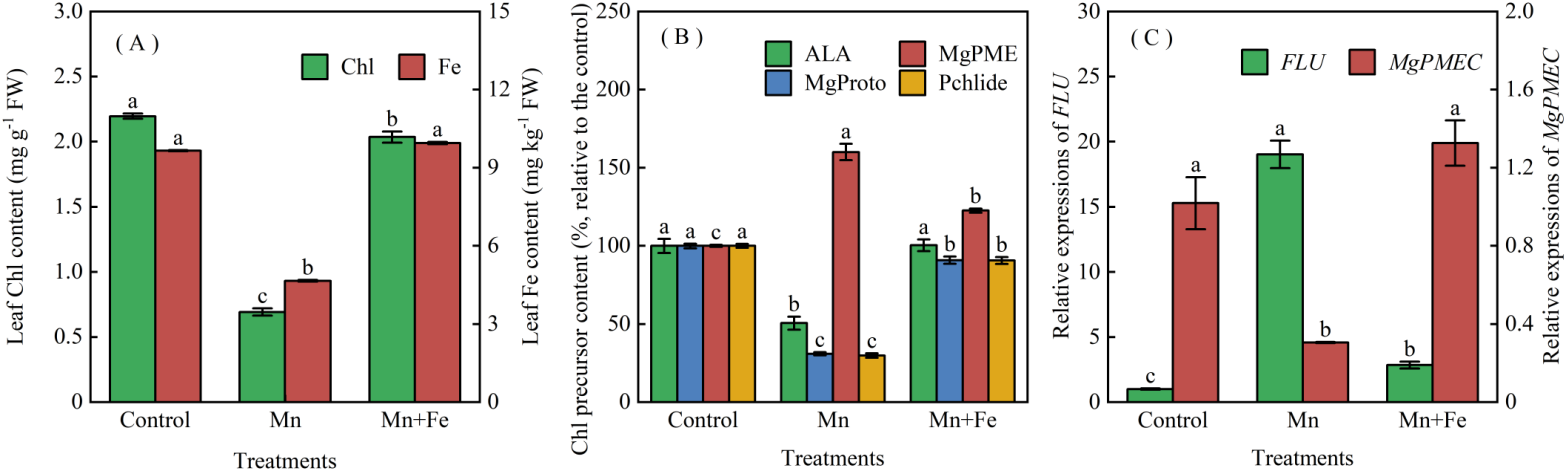
Effects of Fe supplementation on concentrations of **(A)** leaf Fe and chlorophyll, **(B)** chlorophyll precursors, and **(C)** relative expression of *Flu* and *MgPMEC*. Seedlings were grown in nutrient solution under three treatments: control (Con), excess Mn^2+^ (0.5 mmol L^−1^, Mn), and excess Mn^2+^ plus Fe (10 μmol L^−1^, Mn+Fe) for 15 days. Values are means ± SE (n = 3). Different lowercase letters indicate significant differences (*p* < 0.05), as determined by one-way ANOVA with Duncan’s multiple range test (the same for all following figures unless otherwise stated).

Hydroponic experiments showed that Fe supplementation (Mn+Fe) increased leaf Fe concentration by 1.1-fold and Chl concentration by 1.9-fold relative to Mn-only seedlings (**Figure 4A**). Chl levels in Mn+Fe plants were comparable to controls (Con), indicating full reversal of Mn-induced chlorosis.

Mn toxicity inhibited chlorophyll biosynthesis by reducing 5-aminolevulinic acid (ALA) and downstream intermediates, and by causing Mg-protoporphyrin IX monomethyl ester (MgPME) accumulation (Yang et al., 2022. Fe supplementation increased ALA by 1.0-fold, Mg-protoporphyrin IX (MgProto) by 1.9-fold, and protochlorophyllide (Pchlide) by 2.0-fold, while decreasing MgPME by 23.6% (**Figure 4B**). ALA was fully restored to control levels; MgProto and Pchlide recovered to >90% of control levels.

Mn stress suppressed MgPME cyclase (*MgPMEC*) expression and upregulated *FLUORESCENT* (*Flu*), which encodes an inhibitor of ALA biosynthesis. Fe supplementation restored *MgPMEC* expression to control levels and reduced *Flu* expression by 85% relative to Mn-only plants (**Figure 4C**).

### Foliar Fe application alleviates Mn-induced chlorosis in field-grown seedlings

**Figure 5:**
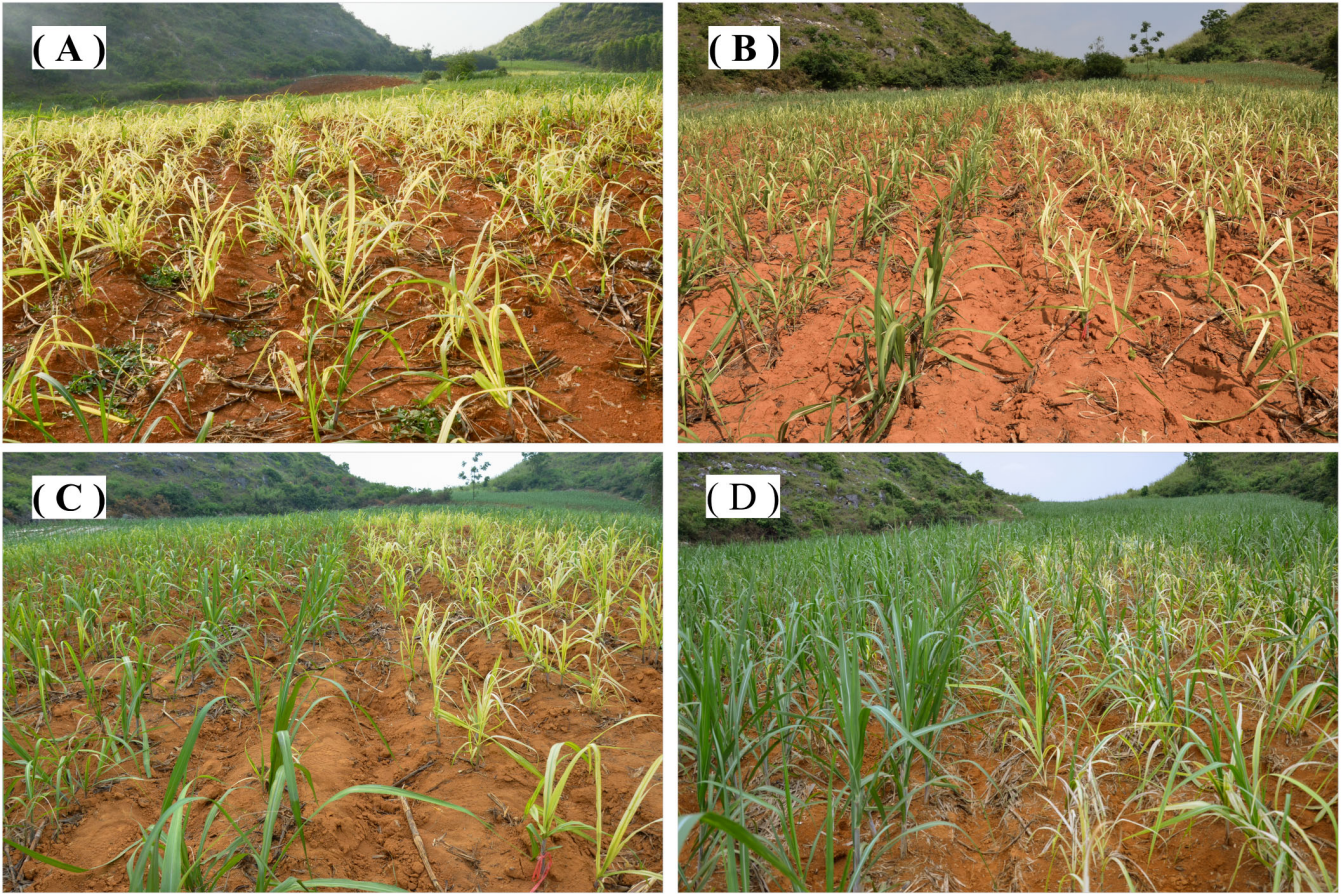
Visual recovery of Mn-induced chlorotic sugarcane seedlings after foliar application with 0.5 g L^−1^ Fe (as FeSO_4_ solutions) at **(A)** 0, **(B)** 3, **(C)** 13, and **(D)** 33 DAT in acidic soil (pH 5.1).

Foliar FeSO_4_ sprays (0.5-1.5 g Fe L^−1^) were applied to chlorotic seedlings (leaf Mn concentration: 360.4 ± 4.9 mg kg^−1^ DW) grown in acidic soil (pH 5.1) in 2024. Similar trials were conducted in 2022–2023 at three additional sites, and the 2024 results are presented as representative. Three days after application, chlorosis symptoms visibly diminished; by 13 days after treatment (DAT), leaves were largely green in Fe-treated plants, whereas control seedlings developed severe chlorosis (**Figure 5**). By 33 DAT, most control plants exhibited extensive chlorosis and necrosis, with some mortality, while Fe-treated plants maintained vigorous growth with darker green leaves, greater leaf number, and increased height.

**Figure 6:**
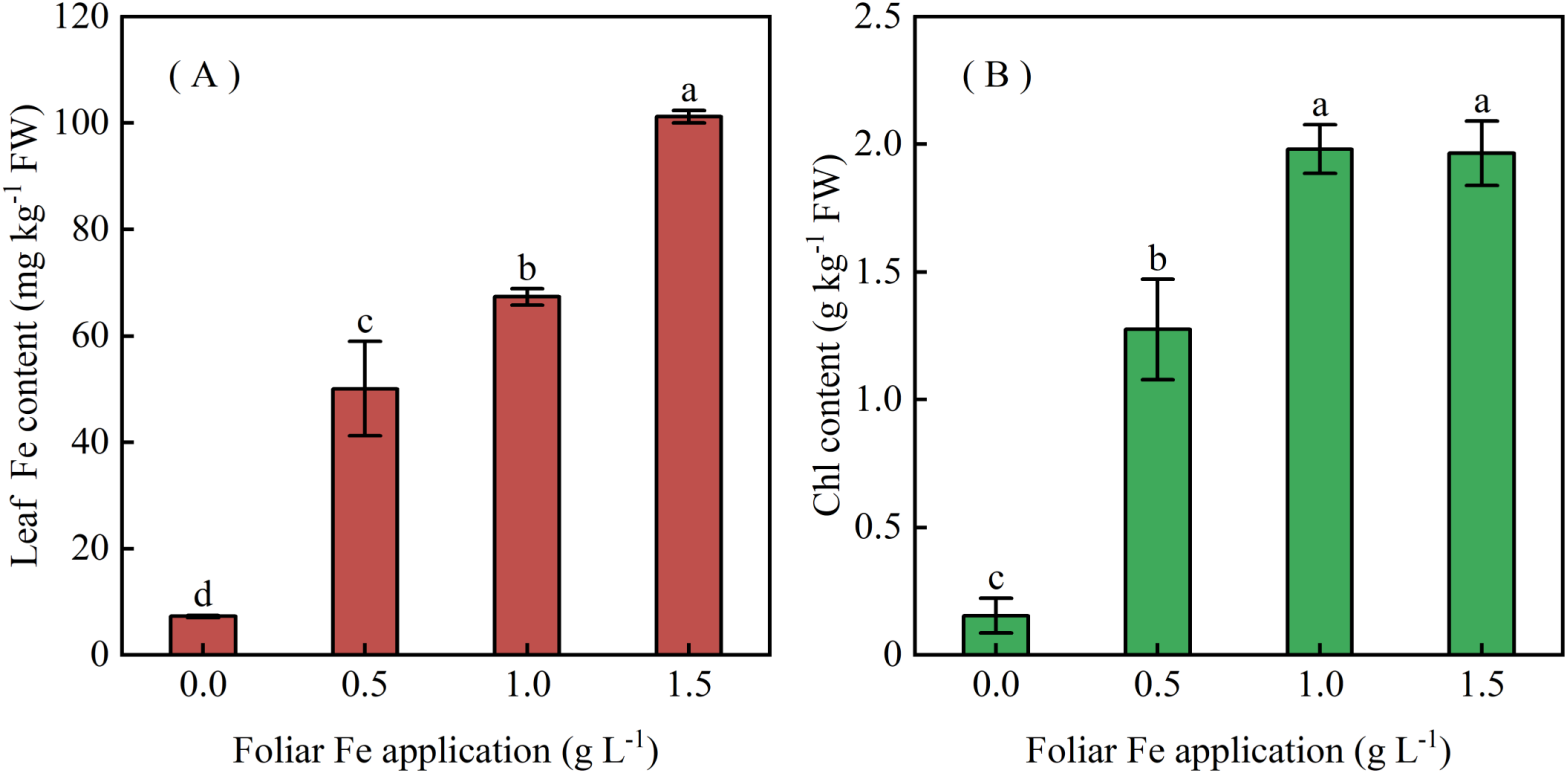
Effects of foliar FeSO_4_ application on **(A)** leaf Fe concentration and **(B)** chlorophyll content in Mn-induced chlorotic sugarcane seedlings at 13 DAT. Plants were sprayed with FeSO_4_ solutions containing 0, 0.5, 1.0, or 1.5 g L^−1^ Fe. Values are means ± SE (n = 3).

**Figure 7:**
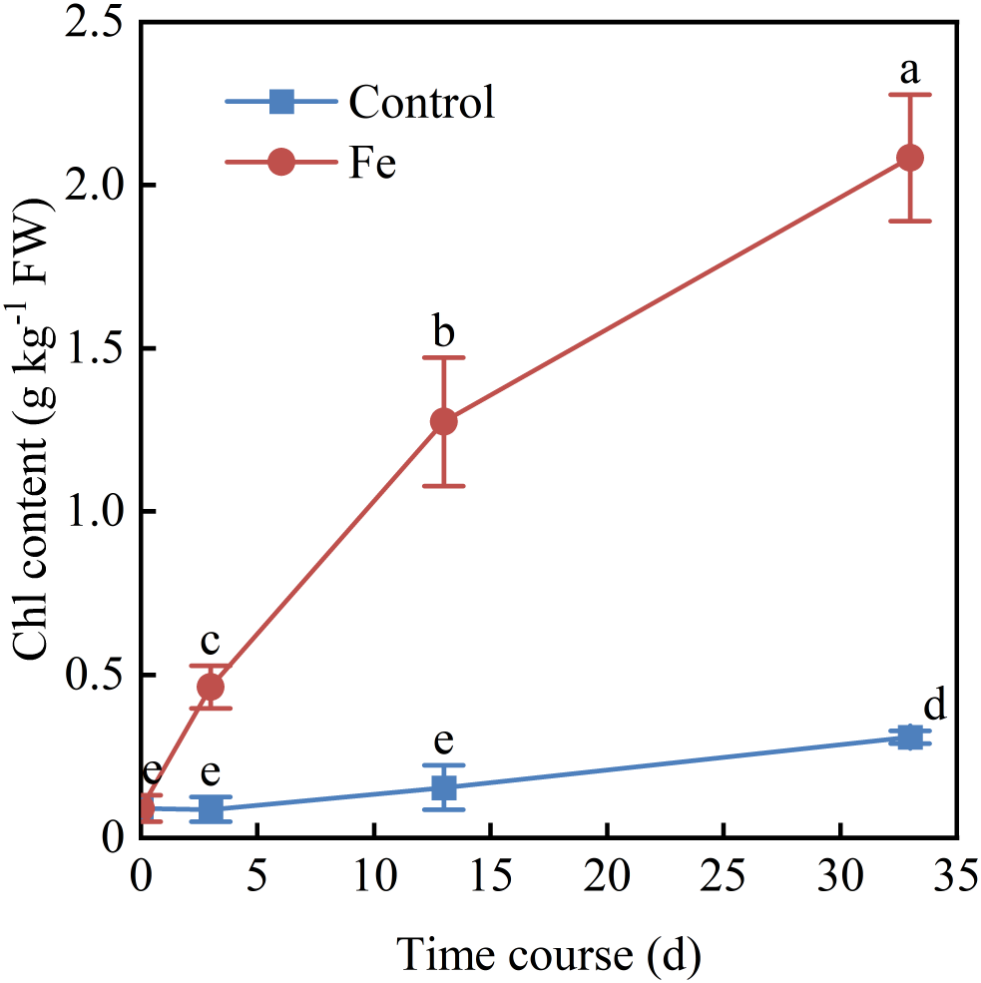
Time course of leaf chlorophyll content in Mn-induced chlorotic sugarcane seedlings following foliar application of FeSO_4_ solutions containing 0.5 g L^−1^ Fe. Measurements were taken at 0, 3, 13, and 33 DAT. Values are means ± SE (n = 3). Different lowercase letters indicate significant differences, as determined by two-way ANOVA by Duncan’s multiple range test (*p* < 0.05).

**Figure 8:**
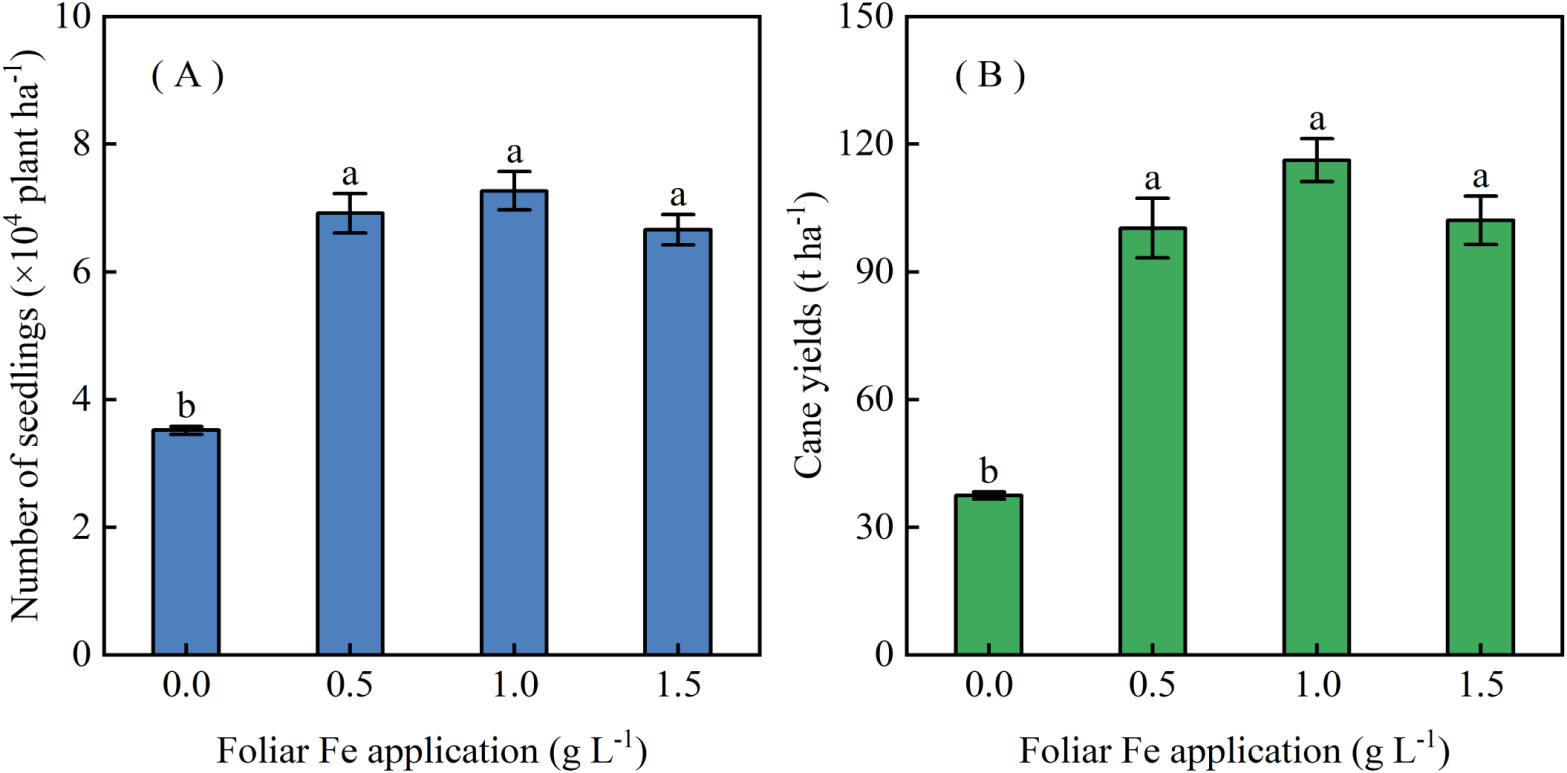
Effects of foliar FeSO_4_ application on **(A)** number of surviving seedlings at 13 DAT and **(B)** mature cane yield in Mn-induced chlorotic sugarcane. Plants were sprayed with FeSO_4_ solution containing 0, 0.5, 1.0, or 1.5 g L^−1^ Fe. Values are means ± SE (n = 3).

Leaf active Fe and Chl concentrations increased with Fe spray concentration at 13 DAT (**Figure 6**), and Chl levels continued to rise until 33 DAT (**Figure 7**). Foliar Fe application also improved agronomic performance: survival plant number increased (**Figure 8A**), and cane yield reached 100.3-116.3 t ha^−1^, 2.68–3.11 times higher than controls (0 g Fe L^−1^), with the highest yield at 1.0 g Fe L^−1^ (**Figure 8B**). These results demonstrate that foliar Fe application is an effective field-level strategy to mitigate Mn-induced chlorosis and enhance sugarcane yield in acidic soils.

## Discussion

### Seasonal recovery of Mn-induced chlorosis in sugarcane seedlings is facilitated by iron derived from rainwater

Previous studies have identified Mn toxicity as the primary cause of widespread chlorosis in ratoon sugarcane seedlings grown on acidic soils in China. However, the developmental progression of chlorosis and its underlying recovery mechanisms remain poorly understood. In this study, we confirmed Mn-induced chlorosis during the dry spring, followed by progressive greening in surviving seedlings and complete symptom remission during the rainy summer. Mechanistic evidence demonstrated that this recovery was facilitated by Fe deposition from rainwater, which counteracted Mn-induced functional Fe deficiency and alleviated the inhibitory effects of Mn toxicity on chlorophyll biosynthesis.

Mn-induced Fe deficiency is a recognized mechanism of Mn phytotoxicity in plants (Foy et al., 1998; Kabir et al., 2016), and visual symptoms can resemble those of Fe deficiency in species such as *Stylosanthes*, pineapple, and sunflower (Blamey et al., 2019; Chen et al., 2015). In sugarcane seedlings, Mn toxicity causes uniform chlorosis in the veins and mesophyll of emerging leaves, with older leaves exhibiting yellowing and whitening (Horiguchi, 1988; Ling et al., 2022). Across 78 surveyed sites, leaf chlorophyll and Fe concentrations were strongly correlated (R = 0.82, p < 0.01; **Figure 1**). Phenological monitoring further showed that chlorotic seedlings accumulated up to 10-fold more foliar Fe by late summer than in early spring (**Figure 2**), coinciding with sharp increases in precipitation (**Supplementary Figure 2**). This seasonal recovery is consistent with reports that chlorotic ratoon sugarcane plants tend to exhibit reduced Mn concentrations after spring, likely due to a dilution effect (Huang et al., 2016). Rainwater application under controlled conditions induced visible greening and increased leaf chlorophyll by 2.7-fold (**Figure 3B**), whereas controls showed progressive albinism. Soil Fe availability remained unchanged (data not shown, p > 0.05), while foliar Fe deposition increased 4.4-fold (**Figure 2B**), suggesting rainwater as the primary Fe source for recovery. The rainwater also contained elements such as N, Mg, Ca, and S. Previous studies have ruled out N, S, or Mg deficiency as causes of sugarcane seedling chlorosis in acidic soils (Huang et al., 2016), supporting the view that Fe is central to Mn-induced chlorosis. However, other seasonal factors such as temperature, light availability, and canopy development also varied alongside seedling greening, and whether these factors contribute independently or interactively to recovery remains to be addressed. Together, these observations suggest that Fe supply plays an important role in the recovery process of chlorotic seedlings.

The biochemical basis of this recovery aligns with our previous findings that Mn-induced chlorosis results from impaired chlorophyll biosynthesis—specifically, inhibition of δ-aminolevulinic acid (ALA) synthesis and reduced conversion of magnesium-protoporphyrin IX monomethyl ester (MgPME) (Yang et al., 2022). Here, Mn stress decreased ALA, MgProto, and Pchlide, while increasing MgPME accumulation (**Figure 4B**), alongside upregulation of the ALA biosynthesis inhibitor *FLUORESCENT* (*FLU*) and downregulation of *MgPMEC* (**Figure 4C**). Fe supplementation restored ALA to control levels, reduced MgPME accumulation, and upregulated *MgPMEC*, while suppressing *Flu* expression by 85%. These results suggest that rainwater-derived Fe facilitates the reactivation of ALA synthesis and relieves the MgPME bottleneck, thus restoration of chlorophyll biosynthesis and potentially enabling complete recovery of chlorotic seedlings by late summer.

### Foliar Fe application as a field-level strategy to alleviate Mn-induced chlorosis

Although Fe supply in growth media is known to reduce Mn uptake and toxicity (Barker and Pilbeam, 2015; Alam et al., 2001; Martínez-Cuenca et al., 2013), field-based evidence for the efficacy of foliar Fe against Mn phytotoxicity has been limited. Our trials provide direct field confirmation that a single foliar Fe spray can rapidly and effectively reverse Mn-induced chlorosis in sugarcane. Visible improvement occurred within 3 days, with complete greenness by 13 days after treatment (DAT) (**Figure 5**). This was accompanied by significant increases in leaf Fe and chlorophyll concentrations (**Figures 6**, **7**), with chlorophyll accumulation showing both dose- and time-dependence, peaking at 2.1 ± 0.1 mg g^−1^ FW at 33 DAT with 1.0 g Fe L^−1^.

Yield recovery under Mn stress has been rarely reported for foliar Fe fertilization (Horst, 2012; Martínez-Cuenca et al., 2013; Barker and Pilbeam, 2015; Huang et al., 2015; Kabir et al., 2016). In this study, a single foliar FeSO_4_ application fully restored yield to levels comparable to healthy plants, achieving up to a 3.11-fold increase over Mn-toxicity controls. The treatment provided sustained benefits throughout the growth cycle, despite the limited phloem mobility typically associated with foliar Fe (Horst, 2012; Barker and Pilbeam, 2015), suggesting that foliar Fe may be more effective against Mn toxicity than against Fe deficiency alone.

Effective Fe spray concentrations vary widely among species, from 0.01 g L^−1^ in strawberry to 1.8 g L^−1^ in pear, pepper, and groundnut (Xiao et al., 2003; Pestana et al., 2012; Roosta and Mohsenian, 2012). In sugarcane, leaf Fe and chlorophyll concentrations increased with Fe spray concentrations from 0.5 to 1.5 g L^−1^; however, survival and final yield did not differ significantly among treatments, indicating that 0.5 g L^−1^ (equivalent to 7.5 g Fe ha^−1^) is sufficient for Mn toxicity mitigation. This foliar approach requires far lower Fe inputs than soil amendments, such as FeSO_4_ applications at 4.5–200.1 kg ha^−1^ for peanut, pepper, and blueberry (Xiao et al., 2003; Roosta and Mohsenian, 2012; Pu et al., 2019), or CaCO_3_ incorporation at up to 5 kg per tree for Mn-toxic woody species (Chatzistathis et al., 2015). These findings highlight foliar Fe application as a resource-efficient, scalable strategy for alleviating Mn-induced chlorosis and improving sugarcane productivity on acidic soils, with substantial advantages in Fe use efficiency and cost-effectiveness over conventional soil treatments.

## Conclusion

Our findings suggest that Fe deposition from rainwater contributes to the seasonal recovery of Mn-induced chlorosis in sugarcane by alleviating Mn-induced Fe deficiency and subsequently reactivating chlorophyll biosynthesis. Importantly, foliar Fe application (optimal at 0.5 g L^−1^) provides an effective mitigation strategy that restores both leaf chlorophyll content and crop yield under field conditions. This study establishes rainwater Fe as a natural remediation pathway but also proposes foliar Fe supplementation as a scalable solution for Mn toxicity in acidic soils.

## Data Availability

The original contributions presented in the study are included in the article/**Supplementary Material**. Further inquiries can be directed to the corresponding author.

## Supplementary material

Supplementary Figure 1 Sequential recovery of Mn-induced chlorotic sugarcane seedlings in a commercial field on **(A)** April 18, **(B)** June 11, and **(C)** July 3, 2013.

Supplementary Figure 2 Cumulative daily precipitation from April 1 to June 30, 2018, in the study area.
