# Supplementary figures and images for: Overcoming Mn-induced chlorosis in sugarcane seedlings by iron
Source: Front Plant Sci. 2026 Jan 22;16:1739211. doi: 10.3389/fpls.2025.1739211 (PMC12872737; doi:10.3389/fpls.2025.1739211)

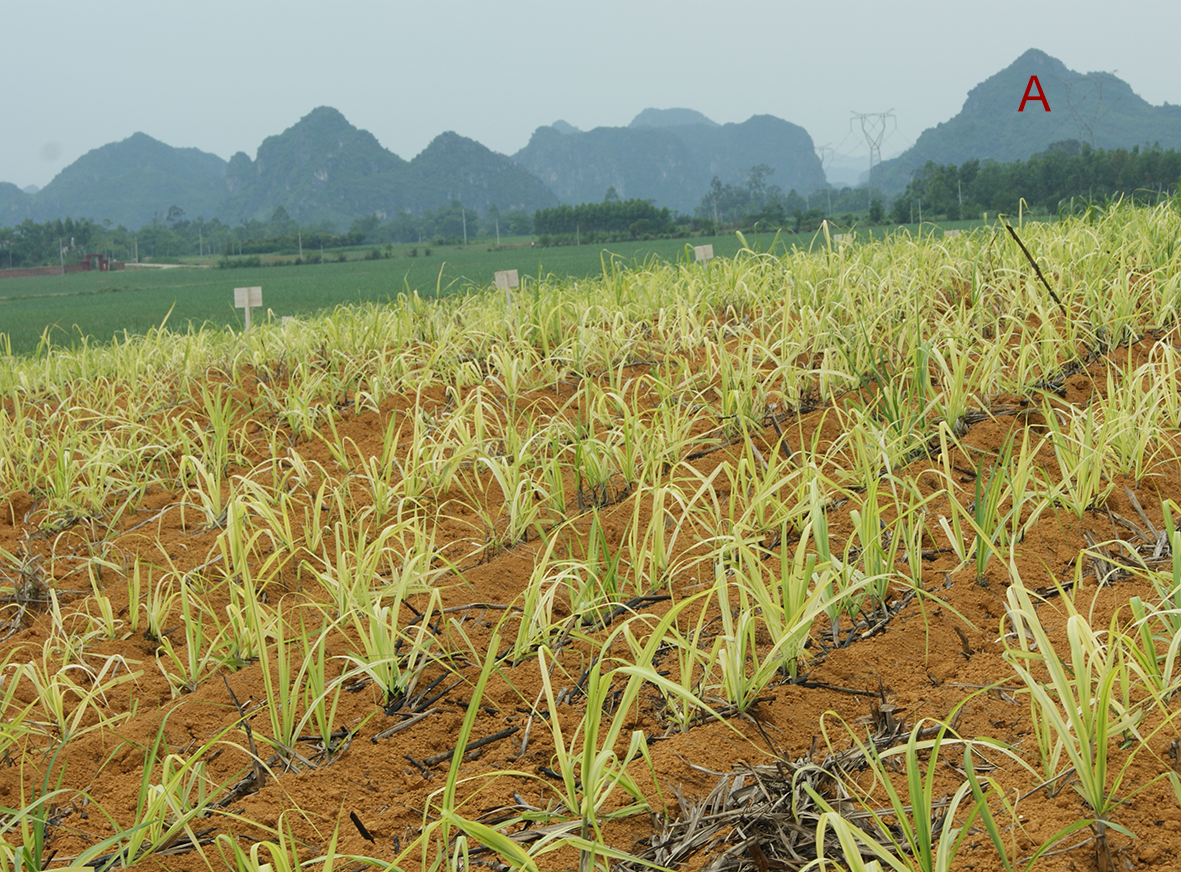

Supplement: Supplementary Figure 1 — Sequential recovery of Mn-induced chlorotic sugarcane seedlings in a commercial field on (A) April 18, (B) June 11, and (C) July 3, 2013. [file Image1.tif]

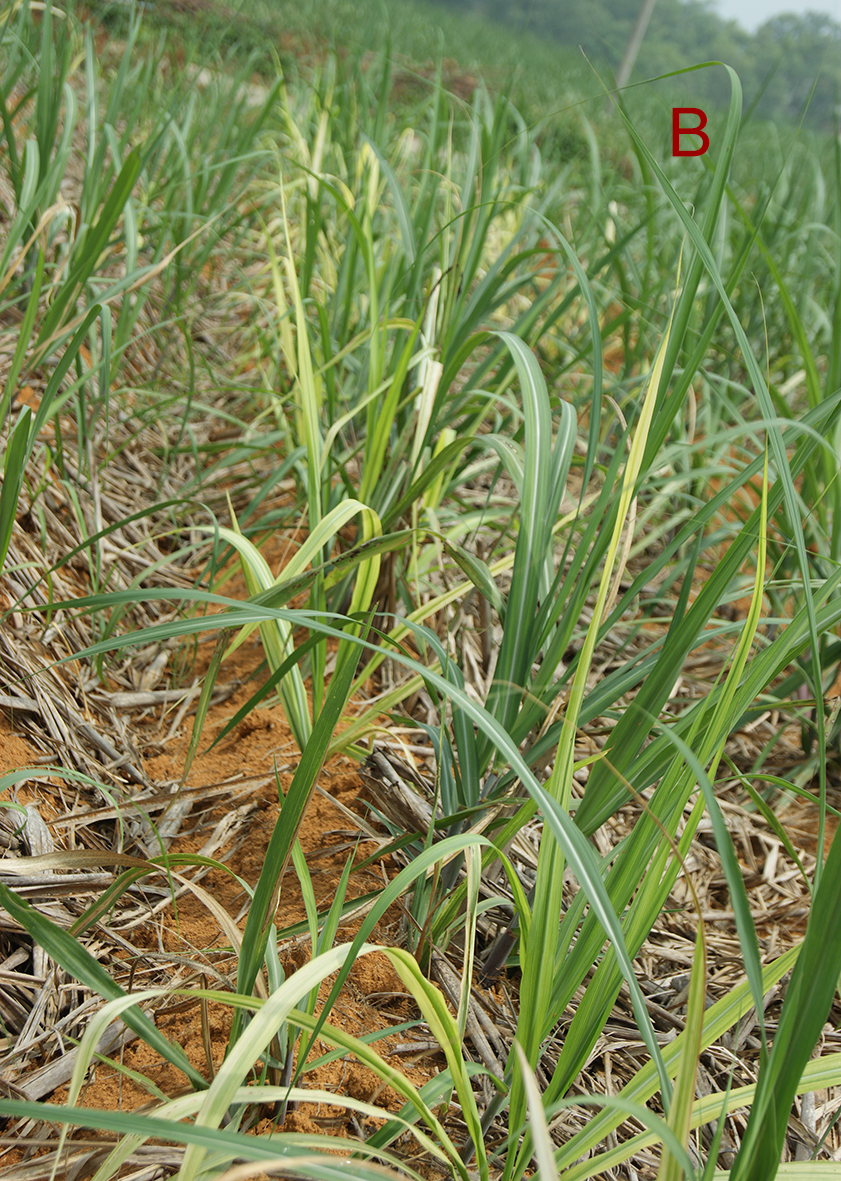

Supplement: Supplementary Figure 2 — Cumulative daily precipitation from April 1 to June 30, 2018, in the study area. [file Image2.tif]

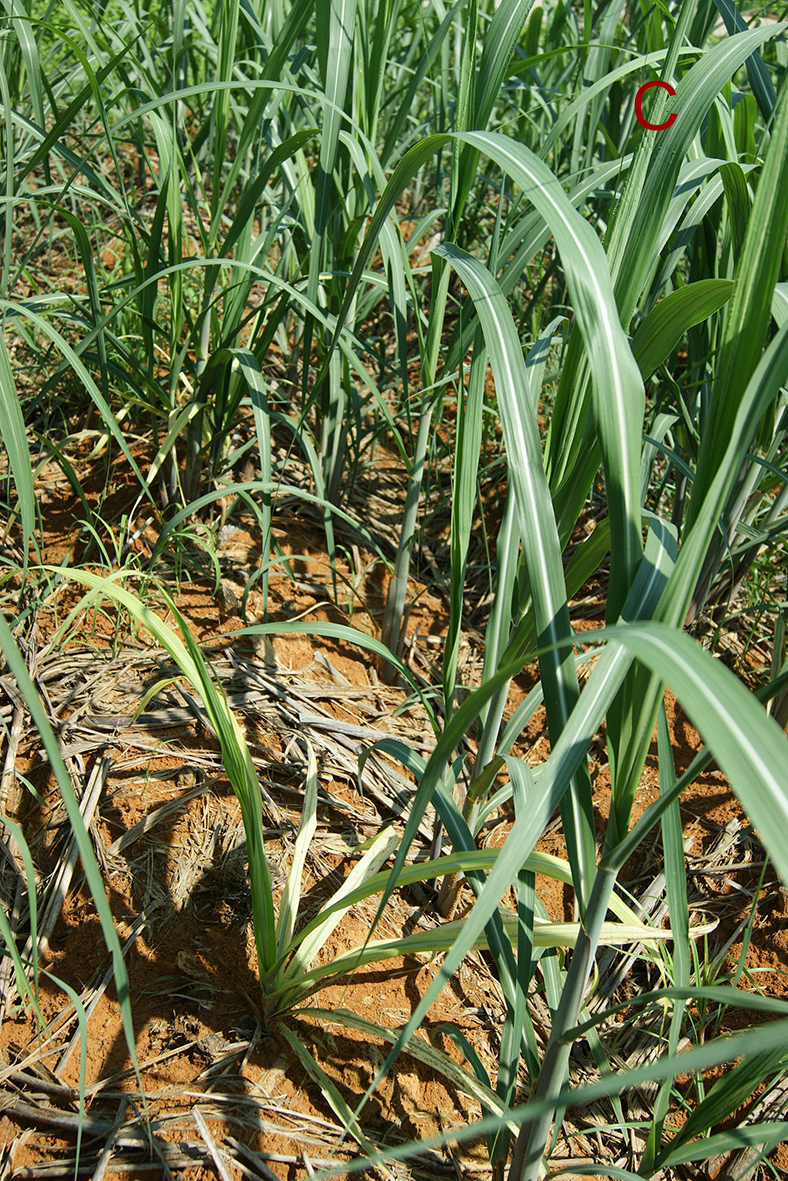

Supplement: Supplementary file 3 [file Image3.tif]

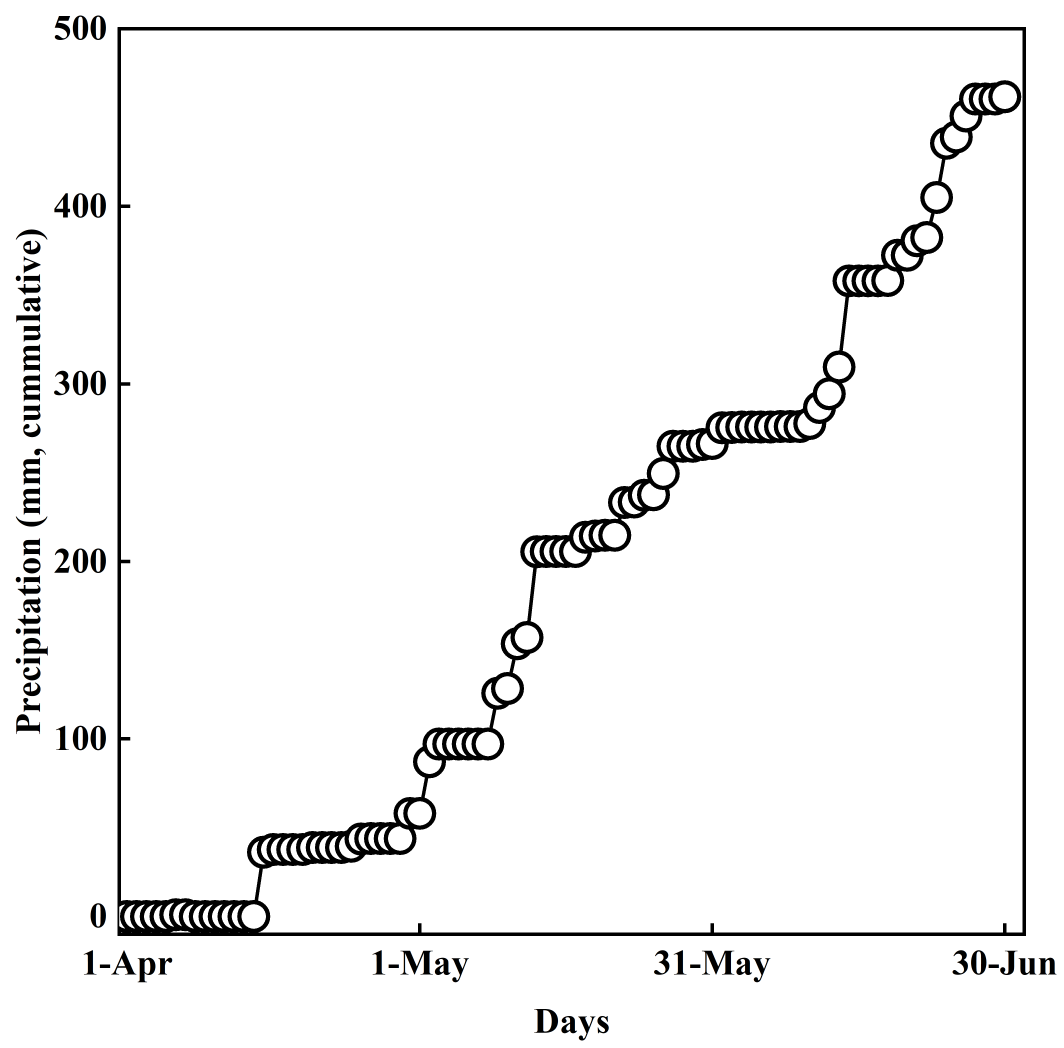

Supplement: Supplementary file 4 [file Image4.tif]
